# COVID-19 pandemic and management of patients with chronic neurological conditions in low-middle income countries: the added burden

**DOI:** 10.11604/pamj.supp.2020.35.2.24186

**Published:** 2020-07-06

**Authors:** Asmaa Hazim, Houda Yacoubi, Houda Guennouni, Jehanne Aasfara, Anas Bennis, Ilham Slassi

**Affiliations:** 1 Mohamed VI University of Health Sciences, Faculty of Medicine, Cheikh Khalifa Ibn Zayed hospital, Neurology Department, Casablanca, Morocco

**Keywords:** COVID-19, neurological disorders, patient management, Healthcare setting organization

## To the Editors of the Panafrican Medical Joursnal

In the Era of SARS-COV-2 disease management, different measures have been proposed emphasizing on reducing risk of contamination, isolating symptomatic patients as soon as possible, setting up separate areas and install barriers to limit contact with patients in triage areas, placing patients with suspected or confirmed COVID-19 in private rooms, protecting healthcare personnel and limiting the numbers of staff providing their care [1]. Nevertheless, the management of non-infected patients within COVID-19-exposed health structures, remained heterogenous particularly in Low-Middle Income Countries where the majority of the available health resources have been allocated to COVID-19 costly management measures and where managing complex chronic patients with multiple comorbidities such as Neurologic patients is already challenging due to the vulnerability of this patient category, scarce qualified human resources and adapted health structures. Indeed, patients with neurological conditions are particularly vulnerable to SARS-COV-2 disease especially patients with neurovegetative impairment, bulbar disorders, myopathic diseases or myasthenia. These patients present an increased risk of contracting SARS-COV-2 disease or developing a severe disease form particularly if the neurological disorders are associated with other comorbidities (i.e. elderly patients, diabetes, hypertension and cardiovascular diseases) or treated by immusuppresive agents (i.e. Multiple Sclerosis Patients) [2]. Recently, the Association of British Neurologists attempted an assessment of risk with COVID-19 from each neurological condition or its treatment. The risk has been subdivided into three levels; low, moderate, and high [3].

**Figure 1:**
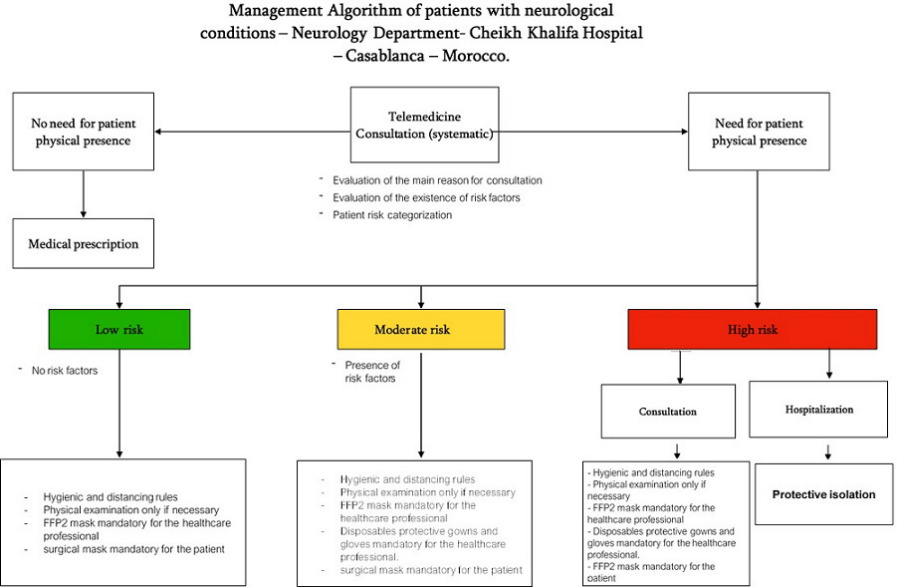
management algorithm of patients with neurological conditions

Based on this risk assessment, we developed an algorithm to improve the management pathway of patients with neurological conditions and help to reduce their exposition level to SARS-COV-2 disease while ensuring continuity of neurological care and without increasing the costs of the already existing preventive measures (Figure 1). In all cases, hygienic and distancing measures applies, the consultation area was redesigned to orientate the patient flow in a one-way direction, the waiting areas were closed, and the cleaning of the consultation rooms was mandatory after each consultation. Telemedicine is definitely an important tool fulfilling the need of protecting vulnerable patients from potential contamination risk by avoiding the necessity of physical presence at the hospital while ensuring an effective clinical follow up. It was the first measure that we have implemented, and which allowed patient triage, remote monitoring of stable patients as well as prescription renewals. However, telemedicine has also some limitation such as breakdown in the relationship between health professional and patient and organizational and bureaucratic difficulties [4], furthermore, in low-middle income countries, the digital divide issue accentuates particularly the barriers of telemedicine implementation as an effective alternative tool. In our case, the telemedicine platform used in our health structure requires the physical presence of the physician in the “telemedicine room” of the hospital. On the patient side, this tool requires installation of a relatively heavy application in the patient´s smartphones as well as high speed internet connection. Alternatively, we experienced the use of WhatsApp® as a substitute to the hospital telemedicine platform, beside the advantage to be a common social media platform suitable with most of the smartphone devices, this application offers also the advantage to be more “user-friendly” and very stable under mobile broadband technology (3G and/or 4G) . In five weeks, we performed over 300 WhatsApp® consultations, received 450 vocal messages and 262 written questions [5].

In our experience, patients with neurological conditions that does not affect their ability to swallow or breath and in whom there is no immune system dysfunction, were not considered to have an additional risk related to COVID-19. Mild or moderate forms of other neurological pathologies such as Parkinson’s disease, multiple sclerosis and epilepsy do not confer neither a higher risk, as long as the ability of the respiratory and swallowing muscles is preserved. When the risk was considered low, the preventive measures adopted consisted in applying hygienic and distancing rules (physical examination only if necessary), obligation of wearing a surgical mask for the patient and FFP2 mask for the healthcare professional. In addition to these measures, healthcare professionals in the Neurology department received the strict instruction to wear protective gowns and gloves when the patient risk was considered as moderate. When the patient´s risk was categorized as high, wearing a FFP2 mask was mandatory for the patient in the consultation area and only if hospitalization was necessary, the patient was isolated and placed in a closed single room. Healthcare professional had the instruction to wear FFP2 masks, disposables protectives gowns, caps and gloves, limitation of visitors to one single visitor with the same dressing rules as healthcare professionals.

## Conclusion

Countries around the globe implemented national comprehensive public health measures in response to the SARS-COV-2 pandemic. The COVID-19 economic impact expected in the near future arise the urge of standardized cost-effective care continuity strategies to protect vulnerable chronic patients especially in Low-Middle Income Countries.
